# Supplementary material for: Effects of Functionalized Iron Oxide Magnetic Nanoparticle Suspensions on Seed Morphology and Physiology in Yellow Maize and Chili Pepper
Source: Plants (Basel). 2025 Nov 25;14(23):3592. doi: 10.3390/plants14233592 (PMC12694257; doi:10.3390/plants14233592)
Supplement: Supplementary file 1 [file plants-14-03592-s001.zip › plants-3927373-supplementary.pdf]

S1. Complementary Characterization of the Nanocomposites

S1.1. Mössbauer Measurements

The room temperature Mössbauer spectrum of the nanocomposite is presented in Fig. S1, while Table S1 presents the hyperfine parameters derived from the fitting of the spectrum with Lorentzian profile lines, by using the least square software MOSF [1]. The spectrum was well fitted with two sextets of magnetite and one doublet attributed to superparamagnetic maghemite (SP-Maghemite). The sextets with hyperfine magnetic fields of 44.1 T and 40.7 T are attributed to ions Fe^{3+} and $Fe^{2.5+}$ of magnetite, respectively, being the intermediate valence 2.5+ confirmed by the isomer shift around 0.6 mm/s exhibited by the second sextet. Although hyperfine magnetic fields of 49.0 and 46.0 T would be expected for ions Fe^{3+} and $Fe^{2.5+}$ of magnetite at room temperature [2], these values can diminish due to surface anisotropy in particles at nanoscale, where the magnetic moments of ions Fe^{3+} and $Fe^{2.5+}$ located on the surface are canted with respect to the mean magnetization inside volume of the particles, significantly reducing the magnetic interactions on surface. The doublet with spectral area of 50% is attributed to ions Fe^{3+} of superparamagnetic maghemite (SP-Maghemite), which is the main phase expected in the nanocomposite due to the easy oxidation of Fe^{2+} to Fe^{3+} during the synthesis process.

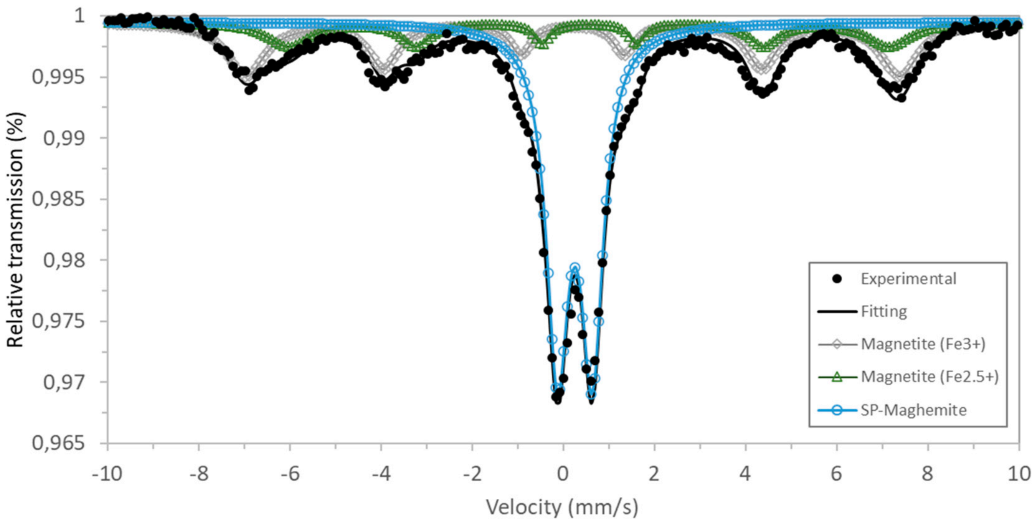

Figure S1. Mössbauer spectrum of the nanocomposite synthesized.

The area ratio R=A($Fe^{2.5+}$)/A(Fe^{3+}) for the sextets of magnetite is 0.79(9), this value being less than the value 1.8 expected for a crystalline and stoichiometric magnetite [3], which can be explained by the presence majority of maghemite in the sample, because the number of ions Fe^{2+} that can have hopping current with ions Fe^{3+} at octahedral sites of magnetite decreases, reducing the spectral area A($Fe^{2.5+}$) with respect to A(Fe^{3+}) and consequently reducing the ratio R.

Table S1 Mössbauer parameters of the iron phases identified in the nanocomposite.

Subspectrum	B_{hf} (T)	δ (mm s^{-1})	2ϵ, ΔE_Q (mm s^{-1})	W (mm s^{-1})	A (%)
Magnetite Fe^{3+}	44.1 ± 0.2	0.32 ± 0.02	0.02 ± 0.02	0.60 ± 0.02	28 ± 2
Magnetite $Fe^{2.5+}$	40.7 ± 0.2	0.62 ± 0.02	0.02 ± 0.02	0.77 ± 0.02	22 ± 2
SP-Maghemite Fe^{3+}	---	0.35 ± 0.02	0.75 ± 0.02	0.63 ± 0.02	50 ± 2

B_{hf} hyperfine magnetic field, δ isomer shift relative to α-Fe, 2ϵ and ΔE_Q quadrupole splitting for sextets and doublets, respectively, *W* line width of the innermost lines of each spectrum, *A* spectral area.

S2. Scripts Used for Statistical Data Analysis with R Software

This section details the scripts used to carry out the statistical analysis of the data collected in the *in-vitro* and greenhouse tests on maize and chili pepper seeds when exposed to different concentrations of iron oxide nanocomposites.

S2.1. Germination Percentage of Maize Seeds at in vitro Level

```
> names(invitro_germinacionMaiz)
[1] "tratamiento" "germinacion"
> factor(tratamiento)
 [1] 0    0    0    c25-3 c25-3 c25-3 c35-4 c35-4
 [9] c35-4 c45-5 c45-5 c45-5
Levels: 0 c25-3 c35-4 c45-5
> anova1 = aov(germinacion~tratamiento)
>  summary(anova1)
              Df   Sum Sq   Mean Sq   F value   Pr(>F)
tratamiento  3     891.7     297.22     4.458    0.0404 *
Residuals    8     533.3     66.67
>  leveneTest(anova1)
          Df  F value  Pr(>F)
group   3    0.3333    0.8018
          8
> shapiro.test(my_data$germinacion)
          W = 0.91231, p-value = 0.2284
>  durbinWatsonTest(anova1)
 lag Autocorrelation D-W Statistic p-value
   1        -0.375                  2.5625       0.936
> TukeyHSD(anova1)
                  diff         lwr          upr            p adj
c25-3-0        6.666667    -14.68235  28.015680  0.7538411
```

c35-4-0	13.333333	-8.01568	34.682347	0.2640524
c45-5-0	-10.000000	-31.34901	11.349013	0.4800522
c35-4-c25-3	6.666667	-14.68235	28.015680	0.7538411
c45-5-c25-3	-16.666667	-38.01568	4.682347	0.1344163
c45-5-c35-4	-23.333333	-44.68235	-1.984320	**0.0329735**

S2.2. Germination Percentage of Chili Pepper Seeds at in-vitro Level

```
> names(invitro_germinacionAji)
[1] "tratamientoAji" "germinacionAji"
> factor(tratamiento)
 [1] 0    0    0    c25-3 c25-3 c25-3 c35-4 c35-4
 [9] c35-4 c45-5 c45-5 c45-5
Levels: 0 c25-3 c35-4 c45-5
> anova1 = aov(germinacionAji~tratamiento)
>  summary(anova1)
              Df   Sum Sq   Mean Sq   F value   Pr(>F)
tratamiento    3    372.9     124.3     0.947    0.462
Residuals      8   1050.0     131.2
>  leveneTest(anova1)
        Df  F value  Pr(>F)
group   3   0.5657   0.6528
        8
> shapiro.test(my_data$germinacionAji)
        W = 0.93088, p-value = 0.3895
>  durbinWatsonTest(anova1)
 lag Autocorrelation D-W Statistic p-value
   1       -0.3280423       2.523810.96
```

S2.3. Root Length of Maize Seeds at in vitro Level

```
> names(invitro_longitudraiz_Maiz)
[1] "tratamiento"         "longitudraiz_maíz"
> factor(tratamiento)
 [1] 0    0    0    c25-3 c25-3 c25-3 c35-4 c35-4
 [9] c35-4 c45-5 c45-5 c45-5
Levels: 0 c25-3 c35-4 c45-5
> anova1 = aov(longitudraiz_maíz~tratamiento)
>  summary(anova1)
        Df Sum  Sq Mean  Sq    F value   Pr(>F)
tratamiento  3      21.92      7.306   1.596    0.265
Residuals    8      36.62      4.578
>  leveneTest(anova1)
        Df  F value    Pr(>F)
group    3    0.8385   0.5098
```

```
           8
> shapiro.test(my_data$longitudraiz_maíz)
W = 0.89436, p-value = 0.1341
>  durbinWatsonTest(anova1)
 lag Autocorrelation D-W Statistic p-value
   1       -0.3862612       2.758885   0.626
```

S2.4. Root length of Chili Pepper Seeds at in vitro Level

```
> names(invitro_longitudraiz_aji)
[1] "tratamiento"          "rootlength_aji"
> factor(tratamiento)
 [1] 0    0    0     25-3 25-3 25-3 35-4 35-4 35-4 45-5
[11] 45-5 45-5
Levels: 0 25-3 35-4 45-5
> anova1 = aov(rootlength_aji~tratamiento)
>  summary(anova1)
              Df Sum  Sq Mean    Sq     F value   Pr(>F)
tratamiento  3          1.670    0.5565   3.415    0.0732
Residuals    8         1.304      0.1630
>  leveneTest(anova1)
          Df F value Pr(>F)
group  3  0.4259 0.7398
          8
> shapiro.test(my_data$rootlength_aji)
          W = 0.91239, p-value = 0.2289
>  durbinWatsonTest(anova1)
 lag Autocorrelation D-W Statistic p-value
   1       -0.04336694      1.953942          0.25
```

S2.5. Seedling Length of Maize Seeds at Greenhouse Level

```
> names(InvernaderoEAFIT_Maiz_alruraPlanta)
[1] "Treatments"          "Seedling Length_Maize"
> factor(Treatments)
 [1] 0    0     0    C25-3 C25-3 C25-3 C35-4 C35-4
 [9] C35-4 C45-5 C45-5 C45-5
Levels: 0 C25-3 C35-4 C45-5
> anova1 = aov(`Seedling Length_Maize`~Treatments)
>  summary(anova1)
                 Df      Sum Sq  Mean  Sq  F value  Pr(>F)
Treatments    3        22.85       7.618       1.135  0.392
Residuals        8  53.71   6.714
>  leveneTest(anova1)
          Df F value Pr(>F)
```

```
group  3  0.1238 0.9434
         8
> shapiro.test(my_data$`Seedling Length_Maize`)
         W = 0.94774, p-value = 0.6042
>  durbinWatsonTest(anova1)
 lag Autocorrelation D-W Statistic p-value
   1       -0.2688047       2.367012   0.722
```

S2.6. Seedling Length of Chili Pepper Seeds at Greenhouse Level

```
> names(InvernaderoEAFIT_Aji_alturaPlanta)
[1] "Treatments"          "SeedlingLength_Chili"
> factor(Treatments)
 [1] 0    0     0    0          C25-3 C25-3 C25-3
 [8] C25-3 C35-4 C35-4 C35-4 C35-4 C45-5 C45-5
[15] C45-5
Levels: 0 C25-3 C35-4 C45-5
> anova1 = aov(SeedlingLength_Chili~Treatments)
> summary(anova1)
              Df Sum  Sq Mean   Sq       F value   Pr(>F)
Treatments   3          0.629    0.2097   0.467     0.711
Residuals    11         4.943    0.4494
>  leveneTest(anova1)
         Df F value Pr(>F)
group  3  0.6808  0.582
> shapiro.test(my_data$SeedlingLength_Chili)
W = 0.94316, p-value = 0.4237
>  durbinWatsonTest(anova1)
 lag Autocorrelation D-W Statistic p-value
   1       -0.2310059       2.386246   0.974
```

S2.7. Stem Thickness of Maize Seeds at Greenhouse Level

```
> names(InvernaderoEAFIT_Maize_grosorTallo)
[1] "Treatments"          "stemthikcness_Maize"
> factor(Treatments)
 [1] 0    0     0    C25-3 C25-3 C25-3 C35-4 C35-4
 [9] C35-4 C45-5 C45-5 C45-5
Levels: 0 C25-3 C35-4 C45-5
> anova1 = aov(stemthikcness_Maize~Treatments)
>  summary(anova1)
             Df Sum  Sq Mean     Sq     F value   Pr(>F)
Treatments   3       1.244      0.4145  3.25       0.0811
Residuals      8       1.020      0.1276
```

```
>  leveneTest(anova1)
          Df F value Pr(>F)
group  3  0.7898 0.5327
          8
> shapiro.test(my_data$stemthikcness_Maize)
          W = 0.96506, p-value = 0.8528
>  durbinWatsonTest(anova1)
 lag Autocorrelation D-W Statistic p-value
   1       -0.2261656       2.06871   0.364
```

S2.8. Stem Thickness of Chili Pepper Seeds at Greenhouse Level

```
> names(InvernaderoEAFIT_Aji_grosorTallo)
[1] "Treatments"          "stemThickness_aji"
> factor(Treatments)
 [1] 0    0     0    0          C25-3 C25-3 C25-3
 [8] C25-3 C35-4 C35-4 C35-4 C35-4 C45-5 C45-5
[15] C45-5
Levels: 0 C25-3 C35-4 C45-5
> anova1 = aov(stemThickness_aji~Treatments)
>  summary(anova1)
              Df    Sum Sq   Mean Sq   F value   Pr(>F)
Treatments   3   0.03184    0.01061     3.032      0.075
Residuals   11 0.03850 0.00350
>  leveneTest(anova1)
          Df F value Pr(>F)
group  3  0.3288 0.8047
          11
> shapiro.test(my_data$stemThickness_aji)
          W = 0.97229, p-value = 0.8904
>  durbinWatsonTest(anova1)
 lag Autocorrelation D-W Statistic p-value
   1       -0.3944444       2.741973          0.51
```

S2.9. Root Length of Maize Seeds at Greenhouse Level

```
> names(InvernaderoEAFIT_Maize_longitudRaiz)
[1] "Treatments"          "rootLength_maiz"
> factor(Treatments)
 [1] 0    0     0    C25-3 C25-3 C25-3 C35-4 C35-4
 [9] C35-4 C45-5 C45-5 C45-5
Levels: 0 C25-3 C35-4 C45-5
> anova1 = aov(rootLength_maiz~Treatments)
>  summary(anova1)
                 Df    Sum Sq  Mean Sq  F value   Pr(>F)
```

```
Treatments   3       55.76     18.59    1.063      0.417
Residuals     8     139.91     17.49
>  leveneTest(anova1)
          Df F value Pr(>F)
group  3  0.1917 0.8991
          8
> shapiro.test(my_data$rootLength_maiz)
          W = 0.9462, p-value = 0.5822
>  durbinWatsonTest(anova1)
 lag Autocorrelation D-W Statistic p-value
   1       -0.2661199       2.422814   0.866
```

S2.10. Root Length of Chili Pepper Seeds at Greenhouse Level

```
> names(InvernaderoEAFIT_Aji_longitudRaiz)
[1] "Treatments"
[2] "RootLength_Chilipepper"
> factor(Treatments)
 [1] 0    0    0    0          C25-3 C25-3 C25-3
 [8] C25-3 C35-4 C35-4 C35-4 C35-4 C45-5 C45-5
[15] C45-5
Levels: 0 C25-3 C35-4 C45-5
> anova1 = aov(RootLength_Chilipepper~Treatments)
>  summary(anova1)
                Df   Sum Sq  Mean Sq  F value   Pr(>F)
Treatments   3     1.673     0.5577     0.955     0.448
Residuals     11    6.423     0.5839
>  leveneTest(anova1)
          Df F value Pr(>F)
group  3  1.0095 0.4252
          11
> shapiro.test(my_data$RootLength_Chilipepper)
      W = 0.93225, p-value = 0.2948
>  durbinWatsonTest(anova1)
 lag Autocorrelation D-W Statistic p-value
   1       -0.5155664       2.944941          0.23
```

S2.11. Root Thickness of Maize Seeds at Greenhouse Level

```
> names(InvernaderoEAFIT_Maiz_GrosorRaiz)
[1] "Treatments"          "RootThickness_Maize"
> factor(Treatments)
 [1] 0    0    0    C25-3 C25-3 C25-3 C35-4 C35-4
 [9] C35-4 C45-5 C45-5 C45-5
Levels: 0 C25-3 C35-4 C45-5
```

```
> anova1 = aov(RootThickness_Maize~Treatments)
>  summary(anova1)
               Df   Sum Sq    Mean Sq  F value  Pr(>F)
Treatments   3    0.04004    0.01335       0.49      0.699
Residuals     8    0.21790    0.02724
>  leveneTest(anova1)
          Df F value Pr(>F)
group  3  0.4604 0.7176
          8
> shapiro.test(my_data$RootThickness_Maize)
W = 0.96882, p-value = 0.8981
>  durbinWatsonTest(anova1)
 lag Autocorrelation D-W Statistic p-value
   1        -0.2651828        2.225779   0.588
```

S2.12. Root Thickness of Chili Pepper Seeds at Greenhouse Level

```
> names(InvernaderoEAFIT_Aji_grosorraíz)
[1] "Treatments"
[2] "RootThickness_Chilipepper"
>  factor(Treatments)
 [1] 0    0     0    0          C25-3 C25-3 C25-3
 [8] C25-3 C35-4 C35-4 C35-4 C35-4 C45-5 C45-5
[15] C45-5
Levels: 0 C25-3 C35-4 C45-5
> anova1 = aov(RootThickness_Chilipepper~Treatments)
>  summary(anova1)
                Df   Sum Sq  Mean Sq  F value   Pr(>F)
Treatments   3    0.2547    0.08489    3.875     0.0409
Residuals    11  0.2409    0.02190
>  leveneTest(anova1)
          Df F value Pr(>F)
group   3  0.4612  0.715
          11
> shapiro.test(my_data$RootThickness_Chilipepper)
W = 0.96777, p-value = 0.8238
>  durbinWatsonTest(anova1)
 lag Autocorrelation D-W Statistic p-value
   1        -0.1981052        2.333055   0.868
> DunnettTest(RootThickness_Chilipepper ~ Treatments, data = my_data)
          95% family-wise confidence level
$`0`
             diff        lwr.ci             upr.ci       pval
C25-3-0  0.24166667  -0.04347217  0.5268055  0.1007
```

```
C35-4-0 -0.07291667  -0.35805550  0.2122222  0.8314
C45-5-0 -0.06916667  -0.37715181  0.2388185  0.8760
>  TukeyHSD(anova1)
        95% family-wise confidence level
Fit: aov(formula = RootThickness_Chilipepper ~ Treatments)
$Treatments
                   diff          lwr          upr         p adj
C25-3-0        0.24166667  -0.07329322   0.5566265581   0.1549281
C35-4-0       -0.07291667  -0.38787656   0.2420432247   0.8962119
C45-5-0       -0.06916667  -0.40936223   0.2710288978   0.9261697
C35-4-C25-3 -0.31458333  -0.62954322   0.0003765581   0.0493000
C45-5-C25-3 -0.31083333  -0.65102890   0.0293622311   0.0767622
C45-5-C35-4  0.00375000  -0.33644556   0.3439455644   0.9999860
```

S2.13. Leaf Length of Maize Seeds at Greenhouse Level

```
> names(InvernaderoEAFIT_Maize_LeafLength)
[1] "Treatments"        "LeafLength_Maize"
> factor(Treatments)
 [1] 0    0    0    C25-3 C25-3 C25-3 C35-4 C35-4
 [9] C35-4 C45-5 C45-5 C45-5
Levels: 0 C25-3 C35-4 C45-5
> anova1 = aov(LeafLength_Maize~Treatments)
>  summary(anova1)
             Df   Sum Sq  Mean Sq  F value   Pr(>F)
Treatments   3     16.84    5.612     1.402     0.311
Residuals    8   32.02    4.003
>  leveneTest(anova1)
        Df F value Pr(>F)
group  3  0.7282 0.5634
        8
> shapiro.test(my_data$LeafLength_Maize)
        Shapiro-Wilk normality test
data:  my_data$LeafLength_Maize
W = 0.98132, p-value = 0.9884
>  durbinWatsonTest(anova1)
 lag Autocorrelation D-W Statistic p-value
   1       -0.4149231       2.542304   0.924
```

S2.14. Leaf Length of Chili Pepper Seeds at Greenhouse Level

```
> names(InvernaderoEAFIT_Aji_LeafLength)
[1] "Treatments"
[2] "LeafLength_Chilipepper"
>  factor(Treatments)
```

```
 [1] 0    0    0    0          C25-3 C25-3 C25-3
 [8] C25-3 C35-4 C35-4 C35-4 C35-4 C45-5 C45-5
[15] C45-5
Levels: 0 C25-3 C35-4 C45-5
> anova1 = aov(LeafLength_Chilipepper~Treatments)
>  summary(anova1)
                Df   Sum Sq   Mean Sq   F value   Pr(>F)
Treatments   3   0.6906      0.23020    2.547     0.109
Residuals    11  0.9943      0.09039
>  leveneTest(anova1)
        Df F value Pr(>F)
group  3  1.1155 0.3845
        11
> shapiro.test(my_data$LeafLength_Chilipepper)
        W = 0.80375, p-value = 0.004109
> kruskal.test(LeafLength_Chilipepper ~ Treatments, data = my_data)
        Kruskal-Wallis chi-squared = 5.7934, df = 3,
p-value = 0.1221
```

S2.15. Dry Biomass of Maize Seeds at Greenhouse Level

```
> names(InvernaderoEAFIT_Maiz_BiomasaSeca)
[1] "Treatments"          "DryBiomass_Maize"
> factor(Treatments)
 [1] 0    0    0    C25-3 C25-3 C25-3 C35-4 C35-4
 [9] C35-4 C45-5 C45-5 C45-5
Levels: 0 C25-3 C35-4 C45-5
> anova1 = aov(DryBiomass_Maize~Treatments)
>  summary(anova1)
               Df   Sum Sq   Mean Sq   F value   Pr(>F)
Treatments   3   106838     35613       3.675    0.0626
Residuals    8      77520     9690
>  leveneTest(anova1)
        Df F value Pr(>F)
group  3  0.3802 0.7701
        8
> shapiro.test(my_data$DryBiomass_Maize)
        W = 0.96504, p-value = 0.8526
>  durbinWatsonTest(anova1)
 lag Autocorrelation D-W Statistic p-value
   1       -0.4630613       2.743959   0.646
```

S2.16. Dry Biomass of Chili Pepper Seeds at Greenhouse Level

```
> names(InvernaderoEAFIT_Aji_biomasaseca)
```

```
[1] "Treatments"
[2] "DryBiomass_Chilipepper"
>  factor(Treatments)
 [1] 0    0     0    0          C25-3 C25-3 C25-3
 [8] C25-3 C35-4 C35-4 C35-4 C35-4 C45-5 C45-5
[15] C45-5
Levels: 0 C25-3 C35-4 C45-5
> anova1 = aov(DryBiomass_Chilipepper~Treatments)
>  summary(anova1)
              Df  Sum Sq   Mean Sq  F value  Pr(>F)
Treatments   3    29.31     9.772     0.362     0.782
Residuals     11   296.83   26.984
>  leveneTest(anova1)
        Df F value Pr(>F)
group  3  0.9476  0.451
        11
> shapiro.test(my_data$DryBiomass_Chilipepper)
        W = 0.90931, p-value = 0.1321
>  durbinWatsonTest(anova1)
 lag Autocorrelation D-W Statistic p-value
   1       -0.08533912      1.850162          0.21
```

References

1. Vandenberghe, R.; De Grave, E.; De Bakker, P.M.A. On the methodology of the analysis of Mössbauer spectra. *Hyperfine Interact* **1994**, 83, 29–49.
2. Mössbauer Effect Data Center. *Mössbauer Mineral Handbook* **2005**, 404.
3. Velásquez, A.A.; Marín, C.C.; Urquijo, J.P. Synthesis and characterization of magnetite-maghemite nanoparticles obtained by the high-energy ball milling method. *J Nanopart Res* **2018**, 20 (72), 1-13.
